# THE TIES THAT RECRUIT, RETAIN, AND BIND: ENGAGING HISPANIC IMMIGRANT FAMILIES IN THE TIME OF CRISIS

**DOI:** 10.1093/geroni/igac059.240

**Published:** 2022-12-20

**Authors:** Jielu Lin, Anna Wilkinson, Laura Koehly

**Affiliations:** National Institutes of Health, Bethesda, Maryland, United States; University Of Texas School of Public Health, Austin, Texas, United States; National Institutes of Health, Bethesda, Maryland, United States

## Abstract

In 2008, we launched Project RAMA (Risk Assessments for Mexican Americans) in Houston, Texas, seeking to understand how multigenerational Mexican immigrant families communicate about familial risk for complex disease. Several lessons were learned. First, our community advisory committee endorsed research goals. Second, we listened to the community with regards to immigration concerns and structural racism. Finally, in the summer and fall of 2008, Hurricane Ike struck the region. Because our team provided support and resources to families in need, we had a higher participation rate post-disaster. Pausing recruitment and postponing assessments led us to unexpectedly discover a long-term intervention effect that was not originally hypothesized. These lessons guide a new initiative focused on Hispanic immigrant families affected by rheumatoid arthritis in Washington DC. We discuss how we address challenges in the on-going project during the Covid pandemic, including recruiting through embedded community clinics and integrating community needs into study design.

